# What E-patients Want From the Doctor-Patient Relationship: Content Analysis of Posts on Discussion Boards

**DOI:** 10.2196/jmir.2068

**Published:** 2012-11-08

**Authors:** Jaqui Hewitt-Taylor, Carol S Bond

**Affiliations:** ^1^Bournemouth UniversityBournemouthUnited Kingdom

**Keywords:** Physician-patient relations, consumer health informatics, eHealth, participatory medicine

## Abstract

**Background:**

People with long-term conditions are encouraged to take control and ownership of managing their condition. Interactions between health care staff and patients become partnerships with sharing of expertise. This has changed the doctor-patient relationship and the division of roles and responsibilities that traditionally existed, but what each party expects from the other may not always be clear. Information that people with long-term conditions share on Internet discussion boards can provide useful insights into their expectations of health care staff. This paper reports on a small study about the expectations that people with a long-term condition (diabetes) have of their doctors using information gleaned from Internet discussion boards.

**Objective:**

The aim of this study was to ascertain what people with diabetes who use Internet discussion forums want from their doctors. The study objectives were to identify what people with diabetes (1) consider their role in condition management, (2) consider their doctor’s role in managing their condition, (3) see as positive elements of their interactions with medical staff, and (4) find problematic in their interactions with medical staff.

**Methods:**

The study used qualitative methodology to explore the experiences, views, and perceptions of individuals participating on 4 Internet message boards. Posts made on the discussion boards were analyzed using the principles of qualitative content analysis. The meanings of sections of data were noted using codes that were developed inductively; those with similar codes were merged into subcategories and related subcategories were combined to form categories.

**Results:**

The key themes identified in the study were ownership of condition management, power issues between people with long-term conditions and doctors, and ways in which people seek to manage their doctors.

**Conclusions:**

People with diabetes valued doctors who showed respect for them and their knowledge, and were willing to listen and openly discuss their options. Patients felt that they could and should take responsibility for and control of their day-to-day disease management. They saw doctors as having a role in this process, but when this was lacking, many people felt able to use alternative means to achieve their goal, although the doctor’s function in terms of gatekeeping resources could create difficulties for them in this respect.

## Introduction

 Management of long-term health conditions is important in terms of costs to health services, and the personal and financial costs to individuals [1]. This includes people receiving the appropriate therapies and how they manage the day-to-day practicalities of their condition.

In many health care cultures, there has been a move away from the idea that health care professionals should take the lead role in how long-term conditions are managed toward encouraging those who are affected to take greater control and ownership of their condition and its management [2]. It has been suggested that greater partnership and sharing of expertise between staff and patients is needed [2]. This changes the relationship that has traditionally existed between health care staff and patients so that how each party sees and enacts their role—and the expectations they have of one another—has become an important part of managing long-term conditions. This study focuses on the expectations that e-patients with a long-term condition (diabetes) have of their doctors.

### Background

Long-term conditions have been defined as conditions that affect a person’s health that cannot, at present, be cured but can be controlled by medication and other therapies [3]. Having a long-term condition affects the individual’s life, but also increases their level of expertise about their health. This has led to the recommendation that people with long-term conditions and health care staff share their expertise to better understand one another’s perspectives and develop treatment regimes that meet clinical needs and accommodate the lifestyles and priorities of individuals [2].

Although the ideal is the sharing of expertise between health care professionals and patients and the development of self-management skills in people with long-term conditions, some issues have required clarification or further exploration. If expertise is to be shared, what knowledge each party is expected to contribute merits debate. The UK Department of Health [2] suggests that people with long-term conditions are experts in how their condition affects them, their social circumstances, and attitudes toward risk, values, and preferences, whereas health care professionals have expertise in the diagnosis, disease processes, prognosis, treatment options, and outcome probabilities. Doubts have been expressed about whether patients can attain expertise in the theory behind disease processes and management [4]; however, there have also been suggestions that people who live with long-term conditions may attain greater medical or technical knowledge of their condition than some health care staff [5,6]. In addition, although the principle of sharing expertise is regularly articulated, whether health care staff accept that patient expertise is as valid and important for condition management as their own has been questioned [4].

The principle of enabling people with long-term conditions to manage their health needs requires an acknowledgment of a person’s right to make their own decisions about their health [3,7]. This may not always be easily enacted in practice, especially when a patient’s views are at odds with those of health care staff [8,9]. How differences of opinion between health care staff and those seeking to take greater control of managing their conditions will be reconciled requires some thought [10,11]. The focus of debates on the development of expert patients has tended to be on how staff view and accept patient expertise, including knowledge of the physiology, pathophysiology, and treatment options related to their condition. Ahmad et al [12] explored the perceptions of doctors toward patients who bring Internet information into consultations. Although negative perceptions were common, doctors were found to have favorable perceptions of “self-educators” (patients with established conditions who used Internet information to support medical visits without challenging the expertise of the doctor).

Less is known about the expectations patients seeking greater control of their health conditions have of medical staff and how they view the contributions doctors make to their condition management. This move of patients taking greater control of their care merits consideration.

People use a variety of information sources to help them take control of managing their health needs, and Internet discussion boards and other networking sites are an increasingly popular source of such information. In the United States, the Society for Participatory Medicine supports the concept of the e-patient [13]. In this instance, the *e* in e-patient, according to “Doc Tom” Ferguson, the founder of e-patients.net [14], refers to patients who are “empowered, engaged, equipped, and enabled.” However, Fox [15] uses the *e* in much the same way as it is used in the term *eHealth*, identifying e-patients as “Internet users who have looked online for health information.” Thus, the term *e-patient* may refer to more than one phenomenon. In this paper, it is applied to people who are diagnosed with a long-term condition (diabetes) who use the Internet to gain information, advice, or support from their peers. There are many Internet forums devoted to specific conditions managed or moderated by health care staff or by people who live with the condition(s) in question that supply a mixture of information, advice, and support. They may be “open access” boards that require a sign-in process to make posts but are readable by anyone using the Internet, or “restricted access” boards that require some form of membership and a sign-in process to both make and read posts. The type of information shared through Internet discussion boards may provide very useful insights into the perspectives of people who live with long-term conditions, including their perceptions of their responsibilities and those of health care staff. The postings may also give an indication of the types of knowledge that people with long-term conditions share with one another and how they view this information.

The discussion boards included in this study were not moderated by health professionals. Previous research has looked at the use of websites and discussion boards from a professional perspective. Glasgow et al [16] conducted a randomized controlled trial that allocated patients with diabetes to an education website or to the website and human support. Richardson et al [17] conducted a randomized controlled trial about increasing physical activity for several groups of patients, including some with Type 2 diabetes. These types of studies place the health professionals at the center of the interaction and includes patients who are not already e-patients by the nature of the research design. Hartzler and Pratt [18] assert that health professionals have little understanding of how information shared by patients compares to their expertise and that this understanding is necessary to underpin the development of peer-support tools. Therefore, this study approached the question from the perspective of patients and focused on people who were discussing their self-management needs with their peers in arenas not managed by health professionals.

### Study Aims

The relative lack of information on persons with diabetes’ expectations of their doctors and the potential value of the information available on Internet message boards led to the research question: What do people with diabetes who use Internet discussion forums (e-patients) want from their doctors?

The objectives of the research were to identify (1) what e-patients with diabetes consider their own role and their doctor’s role in managing their condition, (2) what e-patients with diabetes see as positive elements of their interactions with medical staff, and (3) what e-patients with diabetes find problematic in their interactions with medical staff.

## Methods

Qualitative methods of data collection and analysis were used because the intention was to explore the perspectives of people living with diabetes. The aim of the study was to explore an individual’s perceptions, not to make judgments as to whether the information provided or the recommendations made were right or wrong.

### Method of Data Collection

Analysis of posts made on Internet discussion boards was carried out using qualitative content analysis. All the subjects and threads within the discussion boards selected for the study were examined. The posts and responses to posts deemed to contain relevant data were copied verbatim from the boards into Word documents. These documents were used for further analysis. The selected boards were all moderated and the moderators’ roles included editing and removing posts deemed to be offensive or inflammatory.

When posts were removed from the boards, this was indicated by an annotation from a moderator. This was occasionally seen on all boards included in the study and may have affected the data gathered because some opinions, particularly those that disagreed in strong terms with the views of other participants, were not available for analysis. Although no editing was seen in the posts and responses to posts that were deemed to be relevant to the study, we cannot know whether other deleted posts would have been relevant.

### Sample

Internet discussion boards about diabetes were selected for this study. Diabetes was selected as the long-term condition because a variety of suitable diabetes message boards existed. However, the focus of the research was not on diabetes per se, but on the management of a long-term condition. Eight open access boards moderated by people with diabetes were identified. Boards moderated by diabetic patients rather than health care professionals were selected because it was thought that these might give the most uncensored views of participants’ experiences of health care. The 4 most active boards were selected for this study. Two of the boards were owned by organizations from the United States and 2 were owned by organizations from the United Kingdom, but participants on these boards came from all over the world. All of the posts made onto these 4 boards during November 2010 were included in the analysis.

All the threads on the boards were examined and posts relevant to the study aims were extracted from these threads. A summary showing the numbers of threads relevant to the study for each board, relevant posts within these threads, average number of posts per thread, the number of unique contributors to the discussions across these threads during the study, and the average number of posts relevant to the study made by each poster are presented in Multimedia Appendix 1. Although these were the unique contributors for each board, individuals could contribute to discussions on more than one board. The extent of this is impossible to determine with any certainty because an individual could adopt a different screen name for each board they participated on. However, 5 identical names appeared across all 4 boards. Therefore, although 4 different discussion boards were used, some of the contributors were not unique to each board.

All of the posts made during the study period (November 2010) were included in the study, regardless of the type of diabetes individuals reported having because the focus was on expectations of doctors, not the medical specifics of condition management. Although the term *e-patients* is used to refer to those using the boards in question, one limitation of the sampling frame is that those who posted on the boards may not be representative of the entire population of people with diabetes, or even those who use the Internet as a source of information and support. Those who use the boards, especially those contributing regularly, are likely to be the most vocal of this group.

### Data Analysis

Two researchers used qualitative content analysis to analyze the data. Data were analyzed manually rather than electronically because of frequent use of abbreviations and “web speak.” Data were analyzed inductively and sections of data were coded by meaning. After all the data was coded, sections with similar codes were merged into subcategories; related subcategories were combined to form categories. Each researcher coded the posts from 2 of the 4 boards and then each researcher coded 1 of the other researcher’s boards. The researchers then compared codes and codings. The intention of this cross-analysis was to increase the depth of analysis by having a second coder provide another perspective and ensure that nothing was missed. Where there were differences in the codes used for sections of data, these were discussed and agreement reached. Agreement between the 2 researchers was reached in all cases. The differences in code allocation related to 1 researcher identifying more issues in sections of data than the other, rather than disagreement over the meaning of the data.

### Ethical Issues

Because the study used information posted on open boards, contributions were regarded as being in the public domain [19,20]. It is good practice to anonymize contributions from open message boards when they are used for research purposes to protect the individual’s personal or online identity; therefore, the names and online identities of contributors were replaced by pseudonyms.

Although it is standard practice in qualitative research to use direct quotes to show precise meanings and nuances of a situation, reproducing exact quotes from Internet discussion boards would make it possible to trace an individual’s identity by searching for the quoted phrase. Therefore, some minor changes were made to the quoted messages [21,22]. Key phrases or expressions were kept intact to maintain the meaning of posts, but minor changes were made to “filler” words, some abbreviations were removed, and spelling errors were corrected. The principles of good practice in research using open boards were maintained by not naming the websites used, using pseudonyms rather than user identities, and not using verbatim quotes [23-26].

## Results

The codes and subcategories developed from the data were clustered into the categories (1) ownership of diabetes management, (2) power issues between diabetic patients and doctors, and (3) ways of managing doctors. The findings related to these categories are summarized subsequently with quotes from contributors to the discussions used for illustration.

These categories were developed from written statements, but another category was developed from what was not written: a relative lack of postings about doctors. Although a number of postings referred to doctors and encounters with them, the vast majority of posts, including posts about what might be deemed to be about medical matters, made no reference to (or only passing reference to) medical staff. There was sometimes a suggestion that doctors were a necessary, but not always key, part of diabetes management and that a significant part of their necessity related to how health care systems worked and the gatekeeper role that doctors had rather than their knowledge or expertise. “Barry” summed this up: “Doctors are only useful because they can write prescriptions and order blood tests.”

### Ownership of Diabetes Management

The majority of contributors considered themselves to be responsible for their condition management. This was perhaps why most posts did not refer to medical staff because decision making and responsibility was felt to rest primarily with the individual. This was, for many, the only logical option. For example, “Sarah” posted: “We know and care more about our bodies than anyone else, and have to take responsibility for our own health.”

Although the prevailing opinion was that diabetic patients needed to be in control of their own condition and its management, many contributors valued doctors who worked in partnership with them, learning with and from them. “Jason” commented: “I like a doctor to understand that someone with over 20 years of experience with diabetes might know a thing or two about the condition.”

Within this relationship of mutual learning, what was seen as important was not necessarily that diabetic patients and doctors agreed, but that they were respectful of one another and willing to work together. “Mary” had no problems if she and her doctor had differing opinions: “We may not always agree, but I can trust them, and they trust me. We have an open, frank, and honest relationship.”

When they received information or advice from health care staff, many diabetic patients appeared to use this as a part of, but not the main or even most reliable, aspect of the evidence that they considered in order to decide how to manage their condition. There was a feeling among many contributors that diabetes required the development of self-reliance as well as self-management, and that they learned to rely primarily on themselves to manage the range of information available and decide what advice to follow. “Zena” commented: “You shouldn’t trust anyone except yourself to know about and manage your diabetes.”

However, some contributors felt that medical staff did not encourage self-reliance. Partly for this reason, despite a general consensus that diabetic patients had to make their own decisions, contributors did not always choose to share these with their doctors. “Sharon” explained: “——— reduced my statin dose and I followed his advice. What gives with this distrust of doctors?”

Even those who advocated making one’s own decisions still felt that there were times, especially immediately following diagnosis, when medical advice was vital and should be followed. “Paul’s” advice to a fellow poster: “For now, you need to follow your doctor’s advice and use the insulin dose he prescribes. Later on you can adjust and fine tune your doses to get better and better control.”

One of the complexities of ascertaining how and why contributors chose what information and advice to follow was that although some valued the advice given by medical staff, others did not find the level or type of advice they received helpful or adequate. There was a suggestion that many would have liked more input from their doctors, but developed alternative resources in its absence. Several posts indicated that this was a common problem on initial diagnosis, especially with Type 2 diabetes. Recalling when he was first diagnosed, “Kevin” posted: “I was given a prescription and an instruction sheet, and that was pretty much it.”

This suggestion of a lack of clear guidance or support at a critical point meant that an opportunity for the establishment of a good relationship between diabetic patients and their doctors was lost, and difficult to reinstate. This might account for why in later stages in the course of their disease, many contributors seemed to consider it their responsibility to own and manage their condition, but at the same time suggested that this was not entirely a matter of choice and that they had no option but to do so because of the unreliability of information from health care staff. By choice or by default, they often developed what seemed to be in-depth medical knowledge that they were confident to share with others. “Stephanie” advised a fellow poster: “I’d suggest that you ask your doctor to go off metformin for a while. Type 1s can and do use it, usually if they have insulin resistance issues (indicated by things like high doses or poor insulin action). The benefits of metformin in that situation might be reduced insulin use, less carb spikes, or some other improvement in control.”

This suggestion that diabetic patients often developed what appeared to be medical knowledge was supported by one new board contributor, who posted: “The way you all talk here, you would think you were doctors!”

### Power Issues

Regardless of how knowledgeable or experienced patients were in their diabetes management, an issue for many diabetic patients was that the power lay in the hands of professionals because of their gatekeeping function. This became problematic if their decisions required prescriptions or access to services that doctors did not deem necessary. “Zena” reported asking her doctor for a specific treatment: “My blood sugar has begun to fluctuate more and I am struggling to control it, but when I asked my doctor to prescribe me insulin, he declined and increased my metformin instead.”

Although many diabetic patients saw power as falling unhelpfully in favor of the health care system, some felt they retained the power of choice over which professional they would consult with. “Yasmin” explained: “My advice is to search for endocrinologists in your area, set up meetings, and interview them like you would a potential employee. Find out how they would respond to certain situations and about things that are important to you. It’s OK to decide not to choose a doctor you don’t feel comfortable with.”

Although the idea of selecting doctors was more common where a National Health Service (NHS) did not exist, even within the constraints of the NHS provision, contributors sought and found ways to achieve choice. “Teresa” described how she “...swapped doctors within the practice until I found one that suited me.”

### Managing Health Care Staff

For many diabetic patients, a major aspect of their requisite toolkit for effective condition management was knowing how to manage the medical professionals they encountered. Most people felt that having as good as possible a relationship with doctors was important. “Imogen” posted: “If you are happy with your doctor, it makes a world of difference.”

A part of achieving this good relationship was finding the right doctor. “Stephanie” explained: “It’s kind of first base to get a doctor who accepts and acknowledges what is wrong with you and has some idea of what they are talking about.”

Being clear, confident, assertive, and insistent were skills many considered necessary to effectively manage medical staff. “Millie’s” recommended approach: “Rather than asking, you could just tell them. Asking invites them to offer their opinion. Telling, in a non-confrontational way, does not.”

Having good information and being able to present it was also recommended. “Nathan” explained how he set about making sure he felt an equal of the professionals: “I learned as much as I could about diabetes treatment options and took the time to learn all the medical terminology. Armed with this, I spoke to my family physician and practice nurse and convinced them that I know what I am doing. You need to learn all you can so you can talk to staff on a level standing.”

Preparing for encounters with staff and anticipating their probable responses was advocated because this demonstrated the ability to effectively self-manage. It was sometimes felt to be necessary to be slightly subversive in order to manage health care staff. “Robin’s” approach was not to get into an argument with doctors: “If you are not happy with your doctor’s advice, but can’t change doctor, then listen, smile, and say, ‘Oh, OK.’ And then go and do your own thing anyway.”

Some people shared hints about the practicalities of managing the system and whom it was important to influence in order to get what they felt they needed. “Judy” suggested: “Win the diabetes nurse over with a sensible argument and she will put your point across to the endocrinologist, who trusts her judgment. He will then pass instructions down the line to the family physician”

Although these themes were the key aspects of contributors’ discussions about what made for good and bad encounters with medical staff, there was also an acknowledgment that they themselves influenced the encounter. “Paula” posted: “I usually find that any problems I have with staff stem from my own attitude. If I go in with a negative manner, that’s what I get back.”

Although those who were posting were often clear about their views and had a similar approach to managing their condition, they also acknowledged that their approach might not be the same as that of their peers. “Bill” felt that he and others contributing to the website represented a particular group of diabetic patients: “Too many diabetics are not controlling their condition, so be glad you found this site of knowledgeable people who want to live.”

## Discussion

### Principal Results

The contributors to these boards came from a range of countries. None of the issues identified appeared to be country-specific, although the solutions to problems sometimes were. For example, ways in which medical provision could be accessed or how supplies or prescriptions could be obtained differed, but the issues involved seemed broadly similar. Likewise, although the specifics of diabetes management were different for people with Type 1 or Type 2 diabetes, the issues they raised regarding medical input into their condition management were very similar.

The current ethos in health care is that individuals have the right to make decisions about their health and to be viewed as partners with health care staff in decision making [27,28]. Although many diabetic patients described taking control of, and responsibility for, managing their condition, this did not always extend to a partnership with staff. Many individuals would have appreciated a greater partnership and felt that, although they were responsible for making their own choices, doctors had an important consultative role to enable them to explore their options with a knowledgeable colleague and to make the decision that best met their needs. The problem seemed to be less that diabetic patients did not want medical involvement than that they sought involvement that included equality of status and respect for their knowledge and experience.

The focus of the knowledge that diabetic patients sought recognition of contrasts to much of the literature on expert patients where medical staff are seen as experts on physiology, pathophysiology, and pharmacology, and patients are seen as experts in their own lifestyle, values, and priorities [2,4,29,30]. In this study, there was a suggestion that although doctors did or should have medical knowledge, diabetic patients also had or developed knowledge within what is considered the medical domain. This not only contrasts with the usual perception of how expertise is shared between patients and health care professionals [2,4], but also runs counter to suggestions that patients who are experts in their condition are likely to be more compliant with prescribed treatment [11]. The suggestion from these message boards is that diabetic patients might be more inclined to question medical advice and to seek their own solutions to the medical management of their condition. In the United Kingdom, the Department of Health [2] has long recognized the importance of health care staff respecting and valuing the knowledge of expert patients, but there was a suggestion that the diabetic patient’s expertise, particularly when this included medical knowledge, and especially if it contrasted with medical staff’s views, was not always welcomed.

Although the ideal for many diabetic patients was an egalitarian partnership, they sometimes suggested that the information they gained outside the health care community was superior to that provided by medical staff. This might have been a natural consequence of exploring posts on an online forum because those who had gained adequate information or information that they found acceptable from health care staff might be less likely to use such forums or might be less vocal within the discussions. As Mandana [31] suggests, health care staff giving or perceiving themselves to have given information does not guarantee understanding. In addition, health care staff giving information does not guarantee that it is accepted as valid by those with long-term conditions.

The model that the discussion board contributors described as their ideal is very similar to evidence-based practice. Evidence-based practice is based on the premise that a range of evidence sources are needed to inform practice, including knowledge gained from research sources, other forms of documentary evidence, expertise in practice, the experience of staff, and the experiences and views of patients [32,33]. The difference in the model described by the diabetic patients in this study was that they seemed to place their own research sources, other documentary evidence that they found, their own experience and expertise as the key tenets, with the knowledge, experience, views, and expertise of health care professionals as a separate entity that they considered alongside their own bank of more reliable evidence. Whereas health care staff might question the validity or reliability of a patient’s knowledge [4], diabetic patients often seemed to take this same approach to information offered by medical staff.

Some people with diabetes felt that information and instruction from health care staff would be especially useful in the early stages of their disease. However, initial diagnosis was a time when there was often a perceived lack of advice or guidance from health care professionals, and when individuals turned to other information sources, including other diabetic patients, and began to trust them rather than health care staff. An early opportunity for medical staff and diabetic patients to develop good decision-making partnerships may be lost. This might be a time when a greater focus on developing such a relationship needs to be established.

As well as developing their knowledge and practical skills in diabetes management, diabetic patients described developing skills in managing their interactions with health care staff so as to get the best out of the encounter, often maneuvering their way to achieving the outcome they wanted. This included choosing which decisions or actions they would share so that what they needed or valued from the medical staff’s input was not withdrawn or affected by the choices they made. It has been suggested that empowering individuals with the skills needed to negotiate treatment regimens will encourage positive health decisions and improved outcomes [34]. This study also suggests that people with diabetes see a part of the skills required to achieve improved outcomes as developing skills in managing encounters with staff and negotiating a way to the treatment they feel they need. This often meant that although the relationship between patients and doctors was superficially good, they were unable to be completely open about their approach to condition management, and that doctors remained uninformed about the realities of what did or did not work for individuals.

There appeared to be a complex link between the development of medical knowledge and the tactics that diabetic patients used to get the most out of health care encounters. Some contributors suggested that having this kind of knowledge was a key to being able to negotiate with health care staff, whereas others apparently complied with their doctor’s instructions, but really followed alternative, undisclosed information. These decisions seemed to rest, in part, on how they felt their apparent knowledge and use thereof would affect the outcome of their consultation. What was clear was that diabetic patients felt that having medical knowledge and managing their interactions with health care providers were key aspects of their condition management.

This study showed that some people with diabetes were functioning as effective e-patients where the *e* stands for being empowered, engaged, equipped, and enabled. They showed themselves to be adept at gathering and assessing a range of information from various sources, including medical staff, and making decisions about its relative worth, which did not always fall in favor of the information given by doctors.

### Limitations of the Study

The findings from this study are not intended to be a generalizable picture of the views or experiences of all diabetic patients. The study used a small number of boards and a sample that was chosen primarily for convenience. The findings represent the views of a small number of individuals who may not be typical of the wider population, and may be those who are the most vocal and are less reliant on health care professionals for input about their condition than others are. Some forum users suggested that they were probably more interested in and inclined to take responsibility for the control of their diabetes than many other diabetic patients were, and although there were a high number of posts each day on the boards studied, these were from a small number of individuals in comparison to overall membership numbers. In addition, because some contributors used more than one board, the apparent findings may be skewed by regular, but repeated, postings across boards from a few key individuals. Because the posts from the boards were downloaded once, any that had been deleted by moderators were lost. The patients, however, are among those who have actively adopted self-management, making them early adopters of the movement desired by many health services. As such, the findings are very relevant to practice because the lessons learned from early adopters can be very valuable in supporting diffusion through an adoption curve.

### Conclusion

This study has found that this particular group of e-patients place themselves at the forefront of managing their condition and gather information from peers and professionals in a variety of ways, including through the Internet and in face-to-face interactions. Their expectations and perceptions of health care staff vary, but they do have a baseline set of ideal expectations for their interactions with doctors.

Although diabetic patients did not expect doctors to always agree with them, they did expect to be listened to and respected for their knowledge of diabetes management, both in theoretical and practical terms. They often had or developed knowledge that was within the usual remit of medical staff. When they presented this for discussion, they expected it, and their presentation of it, to be taken seriously and listened to, even if their doctors did not agree with them. In practice, this emphasizes the need for health care professionals to listen to and engage with patients, and to be prepared to discuss information they have gathered and the reasons why this may or may not be applicable, relevant, or helpful in their particular circumstances.

The study suggests there may be a vital point at the time of diagnosis when medical staff and diabetic patients have the opportunity to establish a relationship that can develop into a sound decision-making partnership. However, there is also some evidence in this study that this opportunity is often missed, leading diabetic patients to seek information elsewhere. It may be at this point that decisions about what information considered to have value are made; once made, these decisions or priorities may be hard to reverse. This suggests that early consultations are pivotal in ongoing health care relationships and condition management.

Doc Tom Ferguson’s [14] definition of the *e* in e-patient was for “empowered, engaged, equipped, and enabled.” This study has identified two more:

1. Evaluating. This refers not only to the information e-patients find, but also to the source of that information, be it a Web page, a peer, or a health care professional. It also suggests that this evaluation begins, and trust in sources is established, at an early stage.

2. Equal. The e-patient expects to be an equal member of the team. There is evidence from this study that when this situation is not encouraged by professionals, individuals develop mechanisms to manage situations that place them in a location of equal power, but without the open and honest relationship that is also valued.

This study focused on one condition and used a group of people who may be more interested in self-management or more dissatisfied with their current health care inputs than the average patient is; however, it does introduce some interesting thoughts about the expectations that people with long-term conditions have of doctors and their input into their condition management.

## Multimedia Appendix 1

Summary of the characteristics of boards included in the study.

## Abbreviations

NHS: National Health Service
