# Supplementary material for: What E-patients Want From the Doctor-Patient Relationship: Content Analysis of Posts on Discussion Boards
Source: J Med Internet Res. 2012 Nov 8;14(6):e155. doi: 10.2196/jmir.2068 (PMC3510709; doi:10.2196/jmir.2068)
Supplement: Supplementary file 1 [file jmir_v14i6e155_app1.pdf]

Numbers of threads and posts within threads which were relevant to the study

UK board 1: 38 threads, 279 posts

UK board 2: 45 threads. 627 posts

US board 1: 29 threads. 376 posts

US board 2: 14 threads, 302 posts

Average number of posts per thread

UK board 1: 7.34 posts per thread

UK board 2: 13.95 posts per thread

US board 1: 12.65 posts per thread

US board 2: 23.23 posts per thread

Number of unique contributors per board

UK board 1: 123

UK board 2: 198

US board 1: 146

US board 2: 94

Average numbers of posts relevant to the study made by each contributor

UK board 1: average number of posts per poster: 2.26 (range 1–56, mode 1)

UK board 2: average number of posts per poster: 3.75 (range 1–57, mode 1)

US board 1: average number of posts per poster: 2.57 (range 1–39, mode 1)

US board 2: average number of posts per poster: 3.11 (range 1-15, mode 1)
